# Drug repurposing of ivermectin abrogates neutrophil extracellular traps and prevents melanoma metastasis

**DOI:** 10.3389/fonc.2022.989167

**Published:** 2022-09-05

**Authors:** Hongjun Zhang, XiaoZhu Xu, Rui Xu, Tao Ye

**Affiliations:** ^1^ Department of Ophthalmology, Minhang Branch, Zhongshan Hospital, Fudan University, Shanghai, China; ^2^ Department of Quality Arbitration, Shanghai Institute of Biological Products, Shanghai, China; ^3^ Division of Rheumatology, Huashan Hospital, Fudan University, Shanghai, China; ^4^ Department of Oncology, Minhang Branch, Zhongshan Hospital, Fudan University, Shanghai, China; ^5^ Key Laboratory of Whole-Period Monitoring and Precise Intervention of Digestive Cancer (SMHC), Minhang Hospital & AHS, Fudan University, Shanghai, China

**Keywords:** melanoma, drug repositioning, ivermectin, metastasis, neutrophil extracellular traps

## Abstract

Neutrophil extracellular traps (NETs) have recently been identified to play a crucial role in cancer metastasis. However, the therapeutic target in NETs of melanoma cancer metastasis is still unknown. In this work, we screened a collection of 231 small molecule compounds. We identified ivermectin (IVM), a widely used antiparasitic drug, significantly inhibits neutrophil extracellular traps (NETs) formation after cathepsin B (CTSB) treatment. In vivo, IVM treatment showed no effects of melanoma tumor growth, while the orthotopic melanoma to lung metastasis was significantly suppressed by IVM. Serum level of myeloperoxidase-DNA and neutrophil elastase-DNA were suppressed after IVM treatment. Tumor infiltrated myeloid-derived suppressor cells (MDSCs) were significantly suppressed while tumor infiltrated CD8+T cells in lung was increased after IVM treatment in mouse melanoma model. Mechanistically, IVM targeted a pyroptotic driving factor gasdermin D (GSDMD), and exhibited a Kd of 267.96 nM by microscale thermophoresis (MST) assay. Furthermore, the direct interaction of IVM and GSDMD significantly suppressed GSDMD oligomerization, which are essential for GSDMD-dependent NETs formation. In vitro, treatment with CTSB in bone marrow neutrophils significantly promotes NETs formation, and the release of extracellular DNA was significantly suppressed by IVM pretreatment. Collectively, our results reveal that with the regulation role of IVM in neutrophils and NETs, IVM may potentially be used as a viable therapeutic approach for the treatment of melanoma cancer metastasis.

## Introduction

Melanoma is the cancer of the skin’s pigment cells that caused by abrupt ultraviolet UV radiation from artificial sources or natural sunlight (1, 2). The treatment of melanoma is developing rapidly, including complementary disease prevention and detection strategies. However, in recent decades, the incidence rate of melanoma in white people around the world has increased steadily (3). Although early melanoma is not fatal, it can be cured by surgery (4). The high incidence rate of postoperative melanoma is still manifested by adverse medical history, and the late stage is the difficulty (5). Metastasis of melanoma is regulated by primary tumor cell exfoliation, tumor inflow or expulsion from the blood circulation system, epidermal serum stromal transformation (EMT) and withdrawal of host immune monitoring (6). In recent years, tumor microenvironment (TME) plays an important role in tumor metastasis and helps tumor patients respond (7). New strategies of adaptive immunotherapy, such as antigen-4-related T lymphocyte cytotoxicity (CTLA-4) and programmable cytokine 1 (PD-1), have played an important role in improving the prognosis of melanoma cancer (7). However, the role of innate immune cells in melanoma, especially in cancer metastasis, is still unclear.

In the endogenous immune cells impregnated with TME (such as bone marrow suppressor cells, platelets, macrophages), neutrophils play an important role in the development and metastasis of cancer (8). One of the most important functions of neutrophils is to accept neutrophil extracellular traps (NETs), which is defined as a mechanism to protect neutrophils from bacterial infection (9). Among non-communicable diseases, including gout, diabetes, rheumatoid arthritis, and even 2019 coronary artery disease (10–13), network therapy has received special attention. Recently, people have paid a lot of attention and studied its role in tumor metastasis and immunological deviation (14). Neutrophil infiltration is often found in ulcerative melanoma, and neural stem cells are related to the development of tumor (15). In addition, retrospective metastasis of old neutrophils significantly enhanced melanoma (B16LS9) due to cancer cell metastasis through the NETs (16, 17). Therefore, in our study, we tested the presence of 231 natural small molecule compounds from the database of Drug Bank and found the formation of reticular structures in the primary level lung melanoma metastasis model in mice.

Cathepsin B (CTSB) belongs to the papain family of cysteine proteases from the lysosome and is crucial for a cellular process called lysosomal membrane permeabilization. The function of CTSB was to degrade components of the extracellular matrix, which contributes to the development and metastasis of tumors (18). CTSB is produced constitutively and was identified as a housekeeping protein, and was highly upregulated in malignant tumors (19). Aberrant overexpression of CTSB has been reported in invasive and metastatic cancers including breast cancer, melanoma and colorectal cancer (20). In melanoma, serum level of CTSB were significantly elevated in melanoma patients and identified as a prognosis marker for melanoma mortality (21). Recently, cathepsin B and D are involved in carcinogenesis and constitute bypass loops of the renin-angiotensin system in neck metastatic malignant melanoma cancer (22). In our study, we investigated the precise mechanism of CTSB in melanoma metastatic by controlling NETs formation as well as for screening the suitable molecule to block the above mechanism.

Ivermectin (IVM) is a macrolide antiparasitic drug that consist with 16 membered rings derived from avermectin family members include selamectin, doramectin and moxidectin (23, 24). Currently, IVM is the most successful avermectin family drug and the discovery of the excellent efficacy of IVM against parasitic diseases won the 2015 Nobel Prize in Physiology or Medicine (25). Despite the strong effects on parasites and antiviral effects, a few relevant studies have identified the potential use of IVM as a new cancer treatment. IVM could significantly suppress the mitochondrial membrane potential and inhibit mitochondrial respiration and ATP production in renal carcinoma cells without affecting the normal kidney cells (26). In addition, the proliferation of breast tumor cell lines was significantly suppressed by IVM-dependent cell cycle arrest and apoptosis (27). Therefore, the mechanism of the inhibition of tumor proliferation and the way that IVM induces tumor programmed cell death provide a theoretical basis for the use of IVM as a potential anticancer drug. However, the effect of IVM on immune cells in the tumor microenvironment are not known.

In this study, we found that IVM significantly inhibited the transformation of melanoma We also identified a new mechanism, namely the formation of IVM/GSDMD dependent network, and the recently discovered IVM reconstruction of the micro immune environment for lung tumors. We also explored the possibility of treatment in the context of melanoma metastasis to this pathway.

## Materials and methods

### Cell culture

Bone marrow neutrophils isolated from wide type (WT) mice. Cells were collected in D-Hank’s buffer (1x) of bone marrow from tibias and femurs. Following red cell lysis by ACK, cells were centrifuged (2000 g, 25 min, room temperature) in 62.5% percoll (GE Health care, 17089102) without braking. Neutrophils lay in the bottom of centrifugal tube. After removing supernatants, cells were washed by PBS. The purity of neutrophils was determined by flow cytometry. Cells were cultured in complete RPMI 1640 media (10% FBS, 0.05 mM 2-mercaptoethanol, 1 mM NEAA). B16F10 cells were cultured in complete DMEM media containing 10% FBS.

### Murine models

C57BL/6 mice were purchased from Cyagen. All mice were maintained in pathogen-free facilities in a specific pathogen-free facility at Fudan University. Animal experiments used all female mice with 4-6 weeks. Animals were randomly allocated to experimental groups. All animal experiments were approved by the Institutional Animal Care and Use Committee of Fudan University. The maximal tumor measurements/volumes are in accordance with the IACUC.

### NETs detection with sytox green

NETs formation was quantified using an approach previously described (28). Briefly, bone marrow neutrophils were plated in 96-well culture plate at 3 × 10^4^ per well in RPMI 1640 medium supplemented with 0.5% heat-inactivated FCS and 0.5% BSA. After 8 hours of stimulation, 0.2 μM Sytox green (MCE, HY-K1077) was added for 15 min. Fluorescence was quantified at excitation and emission wavelengths of 488 nm and 520 nm, respectively. Wells were set up in triplicate and the results averaged to obtain a single data point.

### CCK8 analysis of cell viability

B16-F10 cells were seeded in 96 platform plates with 1 × 10^4^ cells/well, after 24 h, treated with mentioned concentrations of DMSO or IVM for 48 h. Replaced the medium with fresh medium, add 10 μl CCK8 solution, and detect the OD_450_ value with a microplate reader after 2-4 h. Data were analyzed and normalized to DMSO control. Viability (%) = [OD_450_ (Cells treated with indicated concentration of drug in the presence of CCK8) - OD_450_ (Only medium)]/[OD_450_ (Cells treated with DMSO in the presence of CCK8) - OD_450_ (Only medium)] ×100.

### Flow cytometry analysis

For neutrophils, bone marrow cells were isolated from C57BL/6 mice after treated with DMSO, IVM (5 μM) for 2 h, washed cells 2 times with PBS, stained with LIVE/DEAD(APC-Cy7) and anti-Ly6G, anti-CD11b for 15 min at 4°C, then washed cells with PBS for 2 times, stained with DCFH-DA for 30 min at 37°C, washed cells with cold PBS for 2 times. BD LSRFortessa was used for data acquisition and FlowJo (Tree Star) was used for data analysis.

For lung FACS analysis, the methods were used as previously described (29). lung was collected, minced and digested by 33.3 U/mL DNaseI (Sigma D4513) and 0.5mg/mL Collagenase IV (Sigma C5138) at 37°C for 1 h. To analyze immune cell in the lungs, cells were incubated with zombie in PBS at 4°C for 30 min in dark. Then, Fc blocking were performed with CD16/CD32 antibodies in FACS buffer (1x PBS with 0.5% FBS, 2 mM EDTA) at 4°C for 30 min in dark. The cells were suspended in FACS buffer with PE-anti-mouse CD8 (clone 53-6.7, Cat#100750), Bv421-anti-mouse CD4 (clone RM4-5, Cat#100544) antibodies APC-cy7-Rat anti-mouse Ly6G (clone 1A8, Cat#127626), APC-anti-mouse Ly6C (clone HK1.4, Cat#128008), Perpcy5.5-anti-mouse CD11b (clone M1/70, Cat#101212), 4°C for 30 min in dark. Flow cytometry was performed by BD Fortessa X20 (BD Biosciences) and Data were analyzed utilizing FlowJo software (Tree Star, Inc.).

### *In vivo* mouse models

To test the effect of IVM on skin melanoma mouse model, B16F10 cells were washed three times with PBS, B16F10 cells (2 × 10^5^) were subcutaneously injected into the dorsal part of mouse (aged 8-10 weeks). After 1 week, mice were randomly divided into two groups, each of 5 mice, and treated as follows: (i) normal diet plus oral administration of 0.5% carboxymethyl cellulose sodium (NaCMC) as control (Vehicle) every day; (ii) normal diet plus oral administration of 10 mgkg^-1^ IVM every day. From day 7, tumor volume was measured every 3 days, and animal survival rate was recorded every day. Tumor volume was calculated as length × width × width × 0.5. Mice with tumor size lager than 20 mm at the longest axis were euthanized for ethical consideration. To analyze effector function of tumor-infiltrating lymphocytes, mice were euthanized on day 21.

### ELISA analysis of serum CTSB assay

The level of CTSB in the serum was quantified using the Cathepsin B. Human ELISA kit (Abcam) in accordance with the manufacture’s protocol. The level of TGF-β, VEGF and MMP9 were analyzed using ELISA kit in accordance with the manufacture’s protocol (Ray-Bio).

### Confocal immunofluorescence imaging

Imaging was performed on a custom modified Olympus FV3000 Laser Scanning Microscope equipped with a × 60 oil immersion lens. Briefly, bone marrow neutrophils were seeded in chambers and stimulated with Control (DMSO), IVM (5 μM), CTSB (5 μM), PMA (20 nM) for 2 h at 37°C. The cells were fixed with 4% paraformaldehyde (PFA) and 0.1% Triton X-100 for 1h at 4°C, followed by 5% BSA blocking for 30 min at 37°C. The cells were gently washed three times with PBS and stained overnight with the indicated antibodies at 4°C. After three washes with PBS, the cells were incubated with the indicated secondary antibody for 1h at 4°C, and gently washed three times with PBS before imaging. The images were analyzed by using Image J.

### Real time-PCR

The RNA from the lung of indicated groups was extracted using TRIzol reagent (Invitrogen). Then 5 ng of mRNA were subjected to cDNA synthesis. RT-PCR was performed using SYBR Green Supermix (Toyobo). The sequences of the primers of the indicated genes are:

**Table:** 

IL-1β	F: 5’-AATGCCACCTTTTGACAGTGA-3’	R: 5’-GTCCTCATCCTGGAAGGTCC-3’
TNF-α	F: 5’-CGAGTGACAAGCCTGTAGCC-3’	R: 5’-ACAAGGTACAACCCATCGGC-3’
IL-6	F:5’-CACTTCACAAGTCGGAGGCT-3’	R: 5’-CTGCAAGTGCATCATCGTTGT-3’
GAPDH	F:5’-TGCACCACCAACTGCTTAGC-3’	R: 5’-GGCATGGACTGTGGTCATGAG-3’

### Western blot

Cells were collected to lysis buffer in cell lysis buffer (CLB) supplemented with protease and phosphatase inhibitor cocktail (Topscience Co., Ltd.) after treated with IVM (5 μM) or CTSB (5 μM). The lysed protein was loaded on a 10% polyacrylamide gel for SDS-PAGE, then transferred to a PVDF membrane at 200 mA for 80 min. The membrane was blocked at room temperature in 5% BSA for 1 h, incubated at 4°C overnight with the following primary antibodies: IL-1β (#12242S), GAPDH (#8884) from Cell Signaling Technology. GSDMD (ab219800), cit H3 (ab5103) was purchased from abcam. Pro-caspase-3 (MA5-32027) was purchased from ThermoFisher SCIENTIFIC. The membrane was washed three times in the TBST and probed with secondary antibodies (Proteintech, China). Finally, the membrane was imaged by imaging system (Tanon, Shanghai, China).

### Protein labeling and microscale thermophoresis analysis

The binding affinity of GSDMD and IVM was measured at 25°C in a binding buffer (PBS containing 0.05% Tween 20) by Microscale Thermophoresis using MONOLITH NT.115. 100 μl His-GSDMD or His-Caspase-4 at a concentration of 160 nM was incubated with 100 μl 100 nM RED-tris-NTA 2nd Generation dye in the dark for 30 min. After incubation, the labeled GSDMD or Caspase-4 was mixed 1:1 with IVM in a two-fold dilution series from 49 nM-150 μM for the measurement. The samples were loaded into NanoTemper Monolith NT.115 glass capillaries and MST carried out using 40% MST power. *K_d_* values were calculated using the mass action equation and NanoTemper software, the data were plotted by the GraphPad Software.

### Compound screening assay

Bone marrow neutrophils were isolated from C57BL/6 mice and cultured in 10% FBS. Cells were pre-treated with murine granulocyte colony-stimulating factor (PeproTech) for 2 h at 5% CO_2_ with 37°C. Neutrophils were preincubated with small molecular compounds (Topscience Co., Ltd.) for 2 h. Then, cells were stimulated with recombinant mouse active CTSB (R&D, Cat#2336-CY) for 24 h at 37°C. NETs were detected by ELISA after staining with the cell-permeable DNA dye Sytox Green.

### Statistical analyses

All statistical analyses were performed utilizing GraphPad Prism 7 (GraphPad Software). Statistical significance was calculated using unpaired Student’s t-tests to compare the means of two groups, one-way analysis of variance (ANOVA) or two-way ANOVA to compare the means of three or more groups. All *p* values are two-tailed, and *p* values < 0.05 are considered significant (*p < 0.05, **p < 0.01 and ***p < 0.001). The data are represented as mean ± S.E.M. or the median with 10 and 90 percentiles.

## Results

### A screen of nature compound system revealed that cathepsin B-induced NETs formation was significantly suppressed by ivermectin

**Figure 1:**
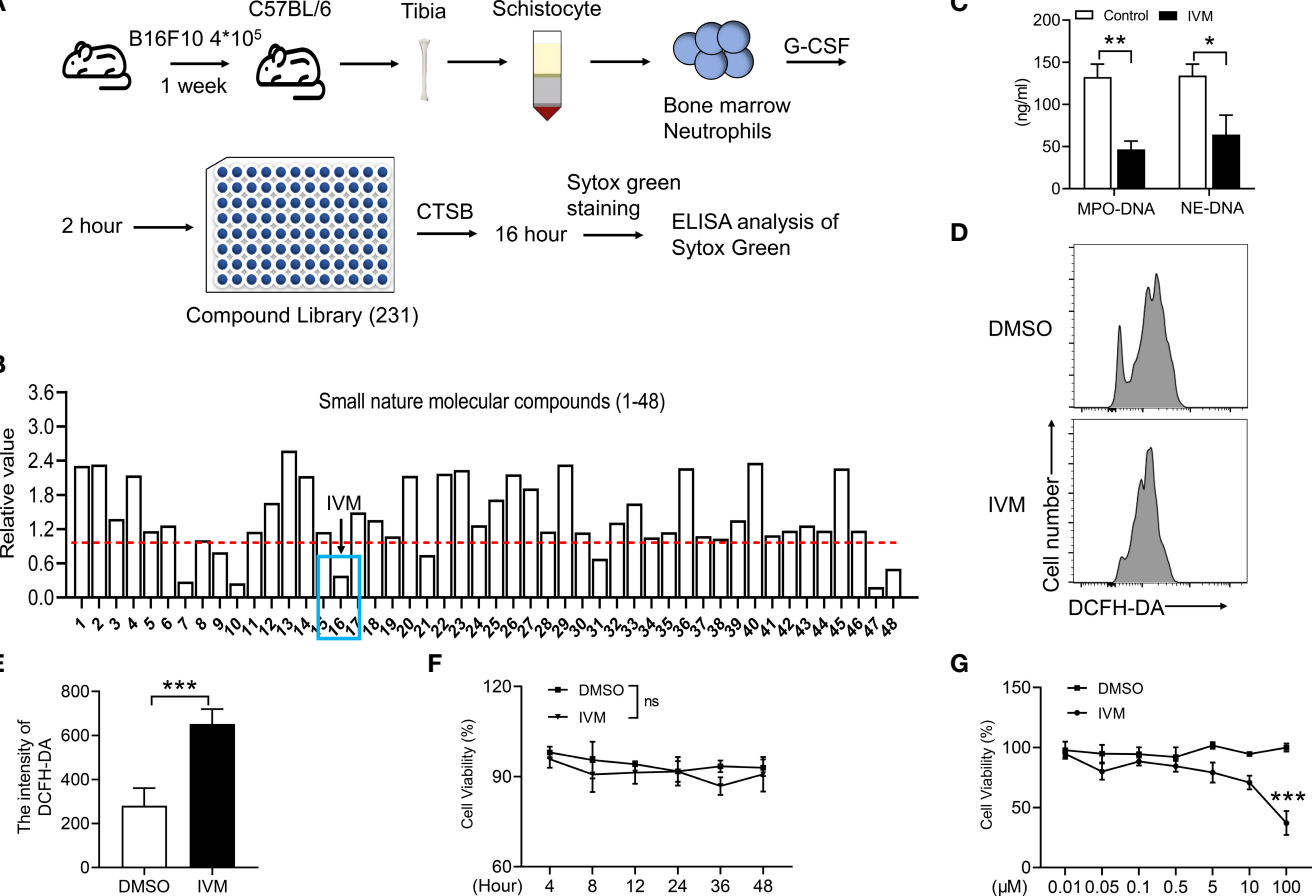
IVM suppressed CTSB-induced NETs formation, which showed no effects on melanoma cell viability. **(A)** Schematic depicting treatment of bone marrow neutrophils for screening of the compound suppressed NETs. **(B)** ELISA analysis of Sytox green in bone marrow neutrophils of NETs. **(C)** ELISA analysis of mitochondrial DNA in bone marrow neutrophils treated with DMSO or IVM (5 µM) for 2 h. **(D, E)** FACS (Fluorescence activated cell sorting) analysis of the DCFH-DA in bone marrow neutrophils treated with DMSO or IVM (5 µM) for 2 h. **(F, G)** CCK8 analysis of cell viability in B16F10 treated with IVM (5 µM) for indicated times **(F)** or indicated concentrations of IVM for 48 h. Data in all panels are representative of at least three [**(C–G)**, n = 3, mean ± SD] independent experiments. *P < 0.05; **P < 0.01; ***P < 0.001; ns, no significance [Significance was examined with two-way ANOVA in **(C)**; Student’s *t*-test **(E)** or two-tailed unpaired Student’s *t*-test **(F, G)**].

It is suggested that drug repositioning is a good strategy for reducing drug development time, lower costs and improve success rates. Drug repurposing are consisting of two strategies including activity-based drug repositioning and computational drug repositioning. Herein, we identified a screen system to detect small molecular compounds that suppressed NETs released after cathepsin B (CTSB) treatment. Bone marrow neutrophils were isolated from a mouse model of melanoma, cells were pretreated with granulocyte colony stimulating factor (G-CSF) for 2 hours. Then the cells were coated on the 96-plate at a density of 2 x 10^4^ with indicated anti-cancer compounds for 48 h (**Figure 1A**). NETs were quantified by the intensity of Sytox green (**Figure 1B**). The results showed the sixteenth compound, ivermectin (IVM), significantly suppressed NETs formation (**Figure 1B**). To further explore the role of IVM on NETs formation. Mitochondrial DNA detected by ELISA. CTSB-induced increased mitochondrial DNA was significantly suppressed by IVM (**Figure 1C**). In addition, several lines of evidence suggest that the formation of NETs are dependent on elevating intracellular reactive oxygen species (ROS) level (30). Our results showed a significant increase of DCFH-DA intensity of bone marrow neutrophils after IVM treatment (**Figures 1D, E**), indicating the role of IVM on ROS production in neutrophils. Furthermore, we detected the different times and concentration of IVM on the cell viability of melanoma cells. The results showed that 5 μM IVM showed no viability effect on B16F10 cells during 48 h (**Figure 1F**). And the viability of B16F10 was only suppressed after a high dosage treatment with IVM (**Figure 1G**). Thus, these findings indicated that IVM suppressed CTSB-induced NETs formation, which showed no effects on melanoma cell viability.

### IVM significantly suppressed melanoma to lung metastasis in a B16F10 *in situ* mouse model

**Figure 2:**
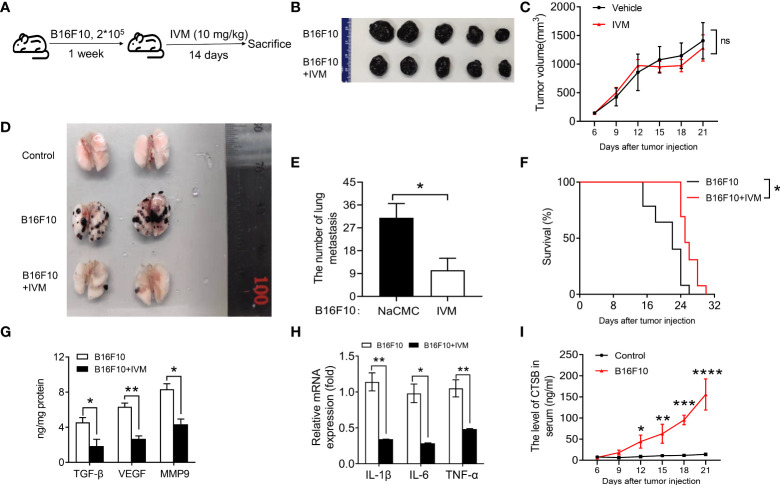
IVM significantly suppressed melanoma to lung metastasis in a B16F10 *in situ* mouse model. **(A)** Schematic depicting treatment of mouse B16F10-bearing melanoma mouse model. **(B)** Images of tumor from IVM (suspended in 0.5% NaCMC) and Vehicle (equal volume of 0.5% NaCMC)-treated mice after B16F10 melanoma inoculation at day 21. **(C)** Quantitative analysis of tumor volume in **(B)** (n = 5 mice per group). **(D)** Images of lung from control, B16F10 and B16F10+IVM groups. **(E)** Quantitative analysis of the number of lung metastasis in **(D)** (n = 5 mice per group). **(F)** Survival curves in IVM- and Vehicle-treated mice after B16F10 melanoma inoculation (n = 5 mice per group). **(G)** ELISA analysis of TGF-β, VEGF and MMP9 in the lung of mice from indicated groups. **(H)** Q-PCR analysis of *IL-1β*, *IL-6* and *TNF-α* in the lung of mice from indicated groups. **(I)** ELISA analysis of CTSB in serum of mouse after B16F10 cells injection. Data in all panels are representative of at least three [**(C, E–I)**, n = 3, mean ± SD] independent experiments. *P < 0.05; **P < 0.01; ***P < 0.001; ****P < 0.0001 [multiple t tests between the two groups in **(E)**; two-way ANOVA in **(C)** and **(I)**; two-way ANOVA in **(G)** and **(H)**; log-rank (Mantel-Cox) test in **(F)**].

We next sought to explore the effect of IVM on melanoma tumor in a murine B16F10 model. We subcutaneously injected 2 x 10^5^ B16F10 cells into each dorsal flanks of C57/BL mice. The mice were then divided into three groups: control mice; B16F10 mice + NaCMC; B16F10 with IVM (10 mg/kg) treatment through oral administration every other day during 14 days (**Figure 2A**). The tumor volume and body weight measurements of the indicated group were taken once 3 days. The results showed that tumor volume was not reduced after IVM treatment (**Figures 2 B, C**). Surprisingly, the metastasis of B16F10 cells into the lung was significantly suppressed after IVM treatment (**Figures 2D, E**). We also detected the survival rate of ovary tumor xenograft mouse model, the survival rate of tumor mice was significantly reduced at 22 days, while the survival rate of tumor mice was still over 50% after 22 days (**Figure 2F**). The level of transforming growth factor (TGF-β), vascular endothelial growth factor (VEGF) and matrix metalloproteinase 9 (MMP9) were significantly increased in lung, while these pro-metastatic factors were suppressed after IVM treatment (**Figure 2G**). To investigate the effect of IVM on immune response. The expression of pro-inflammatory cytokines in lung were detected. In the transcriptional level, cancer related cytokines including *IL-1β*, *IL-6* and *TNF-α* were all reduced after IVM treatment (**Figure 2H**). We also detected the level of CTSB in serum from tumor mouse model. ELISA data showed the level of CTSB in serum was significantly increased after B16F10 cells injection (**Figure 2I**). Collectively, these data indicate that IVM suppressed the progression of melanoma cancer to lung metastasis.

### IVM reshaped the tumor immune microenvironment in lung of melanoma mouse model

**Figure 3:**
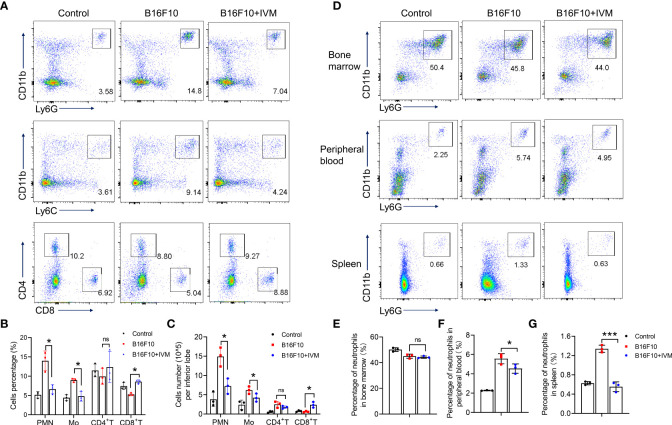
IVM reshaped the tumor immune microenvironment in lung of melanoma mouse model. **(A)** FACS analysis of monocytes (CD11b^+^Ly6C^+^), neutrophils (CD11b^+^Ly6G^+^), CD4^+^ T cells (CD4^+^) and CD8^+^ T cells (CD8^+^) in lung of tissue from control, B16F10 or B16F10 with IVM treatment group mouse at day 21 after melanoma inoculation. **(B, C)** Frequency and cell number analysis of Polymorphonuclear (PMN) neutrophils, monocytes (Mo), CD4^+^ T cells and CD8^+^ T cells from mouse in **(A)**. **(D)** FACS analysis of neutrophils (CD11b^+^Ly6G^+^) in bone marrow (up), peripheral blood (middle), spleen (bottom) from control, B16F10 or B16F10 with IVM treatment group mouse. **(E–G)** Cell percentage analysis of neutrophils of the bone marrow **(E)**, peripheral blood **(F)**, spleen **(G)** from mouse in **(D)**. Data in all panels are representative of at least three [**(A–G)**, n = 3, mean ± SD] independent experiments. *P < 0.05; ***P < 0.001; ns, no significance [two-way ANOVA in **(B, C)**; one-way ANOVA in **(E–G)**].

To further assess the effects of IVM in micro-immune environment in melanoma to lung metastasis. The immune cells in lung were detected by flow cytometry. Our results showed a significant increase of cell percentage of monocytes (CD11b^+^Ly6C^+^) and neutrophils (CD11b^+^Ly6G^+^) in lung of melanoma mice, while a reduced percentage of CD4^+^ and CD8^+^ T cells (**Figure 3A**). And the percentage of monocytes and neutrophils were reduced by IVM while an increased percentage of CD8^+^ T cells after IVM treatment (**Figures 3A, B**). Furthermore, by counting the absolute cell number, we also identified an increase cell number of neutrophils and monocytes in lung of melanoma mice, which were suppressed by IVM. Also, the reduced cell number of CD8^+^ T cells were increased after IVM treatment (**Figure 3C**).

To identified the precise role of neutrophils in melanoma tumor metastasis, we compared the percentage of neutrophils from bone marrow, peripheral blood and spleen. The percentage of bone marrow neutrophils in B16F10 mice was not increased compared with control (**Figures 3D, E**), indicating a no effect on hematopoiesis. The percentage of neutrophils were significantly increased in peripheral blood (**Figures 3D, F**) and spleen(**Figures 3D, G**), suggesting an increased migration of neutrophils from bone marrow. When treated with IVM, the percentage of neutrophils were suppressed in peripheral blood and spleen, while showed no effect on bone marrow neutrophils(**Figures 3D–G**). Taken together, these results demonstrate that IVM suppressed metastasis by reshaping tumor immune microenvironment.

### The formation of NETs in lung of melanoma mouse model was suppressed by IVM

**Figure 4:**
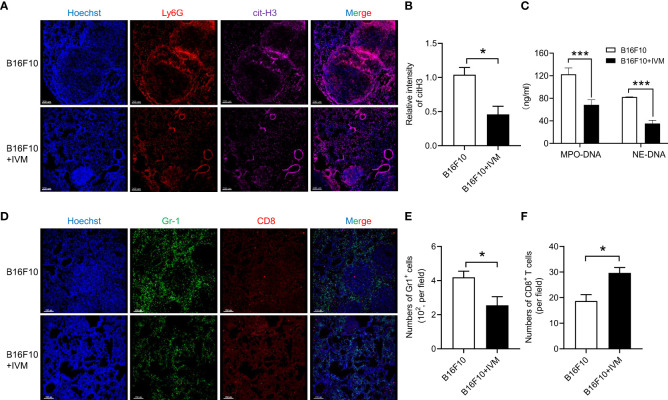
IVM suppressed NETs in melanoma metastasis. **(A)** Confocal analysis of Ly6G (red), cit-H3 (purple) in lung section from B16F10 or B16F10 with IVM treatment group mouse. **(B)** Quantitative analysis of relative intensity of cit-H3 (n = 3 field). Scale bars, 200 µm. **(C)** ELISA analysis of mitochondrial DNA in serum from B16F10 or B16F10 with IVM treatment group mouse. **(D)** Confocal analysis of Gr-1 (green), CD8 (red) in lung section from B16F10 melanoma bearing mouse. **(E, F)** Quantitative analysis of the number of Gr-1^+^ cells **(E)** and CD8^+^ T cells **(F)** (n = 3 field). Scale bars, 150 µm. Data in all panels are representative of at least three independent experiments [**(B, C, E, F)**, n = 3, mean ± SD]. *P < 0.05; ***P < 0.001 [multiple t tests between the two groups in **(B)**, **(E)** and **(F)**; two-way ANOVA in **(C)**].

To explore the role of NETs on melanoma metastasis, we detected NETs by Ly6G and cit-H3 (a biomarker of nuclear DNA decondensing and NETs) in lung section from B16F10 mice. The results of immunofluorescence showed an increased cit-H3 positive staining of Ly6G in lung of B16F10 mice, indicating the formation of NETs. And the increase of NETs was significantly suppressed after IVM treatment (**Figures 4A, B**). Notably, we used a previous report method to detect NETs in serum from B16F10 mice. The results showed the level of mitochondrial DNA was significantly reduced by IVM (**Figure 4C**). It is well known that the activation of CD8^+^ T cells in tumor micro-environment was significantly suppressed by myeloid derived suppressor cells (MDSC) (31). We further investigated the quantity and distribution of MDSC (Gr-1) and CD8^+^ T cells in lung. As expected, immunofluorescence showed a reduced cell number of MDSC in metastatic tumor of lung after IVM treatment, while increased CD8^+^ T cells in lung metastatic tumors (**Figures 4D–F**). Taken together, these results demonstrate that IVM suppressed NETs and reshaped tumor immune microenvironment in melanoma metastasis.

### CTSB-dependent NETs was significantly suppressed after IVM treatment

**Figure 5:**
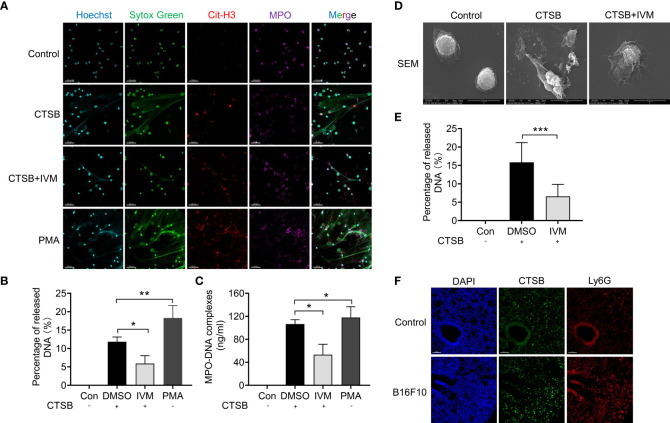
IVM significantly suppressed CTSB-dependent NETs formation. **(A)** Confocal analysis of Sytox Green (green), Cit-H3 (red) and MPO (purple) in bone marrow neutrophils from wide type mice and treated with recombinant CTSB, CTSB with IVM or PMA after 12 h. **(B)** Percentage of released mitochondrial DNA in **(A)** was quantified (n = 3 field). Scale bars, 50 µm. **(C)** Quantitative analysis of mitochondrial DNA in the cultured medium. **(D)** SEM (scan electron microscopy) and quantitative analysis **(E)** of extracellular mitochondrial DNA induced by CTSB in bone marrow neutrophils after treated with IVM. **(F)** Confocal analysis of the expression of CTSB (green) and Ly6G (red) in lung of B16F10 mice. Data in all panels are representative of at least three independent experiments. *P < 0.05; **P < 0.01; ***P < 0.001 [one-way ANOVA in **(B)**, **(C)** and **(E)**].

To further investigate the role of CTSB on NETs formation, we isolated bone marrow neutrophils from WT mice and treated with recombinant CTSB. Confocal analysis showed there is an increased release of NETs (as detected by Sytox green, cit-H3 and MPO positive staining) after 12 hours (**Figure 5A**). The treatment with PMA (20 nM) was showed as a positive control, which showed a highly increased NETs formation (**Figure 5A**). and the results showed nearly no spontaneous NTEs release after IVM treatment (**Figures 5A, B**). Furthermore, we also detected the level of NETs in cultured medium by mitochondrial DNA. The results showed an increased level of mitochondrial DNA in cultured medium after CTSB treatment, and this increase was reduced after IVM treatment (**Figure 5C**). To further explore CTSB-induced NETs formation, scan electron microscopy (SEM) was used to identify extracellular mitochondrial DNA form bone marrow neutrophils. The results showed CTSB-induced NETs was significantly suppressed by IVM (**Figures 5D, E** **)**. In addition, we detected the expression of CTSB through immunofluorescence. Confocal analysis showed that the expression of CTSB was significantly increased in lung of B16F10 mice (**Figure 5F**). Thus, our results showed IVM significantly suppressed CTSB-dependent NETs formation.

### IVM directly interact with gasdermin D and promotes GSDMD oligomerization in neutrophils

**Figure 6:**
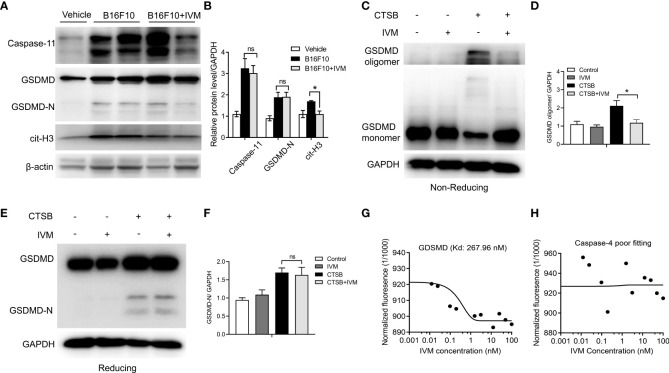
IVM directly interact with GDSMD to promote its oligomerization, and subsequent GSDMD-dependent NETs. **(A)** Western blot analysis of Caspase-11, GSDMD and cit-H3 expression in bone marrow neutrophils from control, B16F10 and B16F10 with IVM group. **(B)** Quantitative analysis of caspase-11, GSDMD-N and cit-H3 in **(A, C)** Non-reducing western blot analysis of GSDMD in bone marrow neutrophils after CTSB or CTSB with IVM treatment. **(D)** Quantitative analysis of GSDMD oligomer. **(E)** Reducing western blot analysis of GSDMD in bone marrow neutrophils isolated from WT mice treated with CTSB for 12 h followed by treatment with IVM for 2 h. **(F)** Quantitative analysis of GSDMD-N. **(G)** and **(H)** MST analysis determined the *K*_d_ of IVM towards His-GSDMD (267.96 nm) **(G)** or His-Caspase-4 (poor fitting) **(H)** labeled with RED-tris-NTA 2nd Generation dye. Concentration is reported in nanomolar. Data in all panels are representative of at least three independent experiments. *P < 0.05; ns, no significance [two-way ANOVA in **(B)**, one-way ANOVA in **(F)**].

We further explore the precise mechanism of IVM on NETs formation. Previous studies have suggested that the activation of Gasdermin D (GSDMD) is required for the generation of NETs (32, 33). However, whether GSDMD activation and GSDMD-dependent NETs is involved in melanoma metastasis are unknown. Bone marrow neutrophils were isolated from control and B16F10 mice. Western blot showed the detection of GSDMD-N terminal in B16F10 mice, but not control mice, indicating the activation of GSDMD (**Figure 6A**). The expression of caspase-11 and cit-H3 was also increased compared with control mice (**Figure 6A**). IVM significantly suppressed the expression of cit-H3 while the expression of caspase-11 and GSDMD-N were not comparable with B16F10 mice (**Figures 6A, B**).

**Figure 7:**
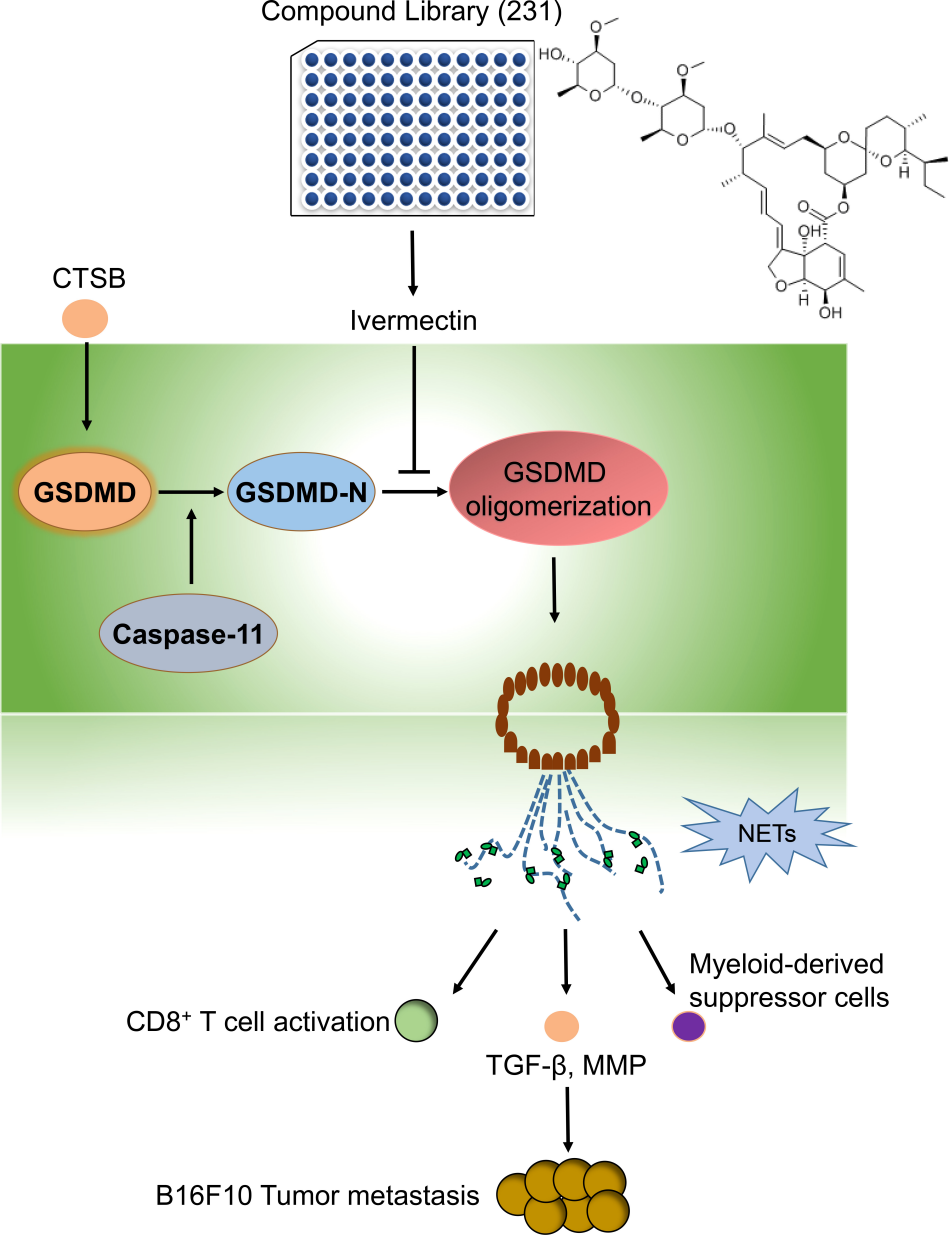
The graphic abstract in this study. By using a compound library, we identified a natural compound IVM, which significantly suppressed CTSBinduced NETs. The precise mechanism of IVM was targeting GSDMD and significantly suppressed GSDMD oligomerization. Thus IVM suppressed GSDMD-dependent NETs. The formation of NETs significantly promotes melanoma cancer metastasis through increasing TGF-b, MMP9 and myeloid-derived suppressor cells and suppressed CD8+T cells activation.

The oligomerization of GSDMD was required for its membrane pore formation (34). We next investigated the effect of IVM on GSDMD oligomerization in bone marrow neutrophils. Bone marrow neutrophils were isolated from WT mice and the cells were treated with CTSB for 12 h. Unchanged western blot showed that CTSB significantly promotes GSDMD oligomerization, while this increase was suppressed by IVM (**Figures 6C, D**). However, the cleavage of GSDMD was not reduced as GSDMD-N showed the same level after IVM treatment. This result suggests that IVM promotes GSDMD oligomerization but not cleavage (**Figures 6E, F**). We measured the binding dissociation constants (*Kd*) of purified GSDMD protein with IVM using microscale thermophoresis (MST). IVM interacted with purified GSDMD protein with a *Kd* of 267.96 nM (**Figure 6G**), while the interaction between IVM and GSDMD showed poor fitting (**Figure 6H**). Taken together, these results indicated that IVM directly interact with GDSMD to promotes its oligomerization, and subsequent GSDMD-dependent NETs (**Figure 7**).

## Discussion

In this study, we report that IVM, a generic drug approved by FDB, has antibacterial and anti-melanoma activities We further identified the precise molecular target of GSDMD for IVM The current research results show that IVM inhibits the formation of GSDD dependent pores and the thermal swelling of cells, which significantly reduces the formation of reticular structures induced by CTSB, leading to melanoma metastasis.

Collectively, our research has found a new potential IVM target in the clinical treatment of melanoma.

Recently, drug repurposing has become a powerful tool for discovering and developing novel anticancer drug candidates (35, 36). Our *in vitro* screening system showed a broad-spectrum anti-parasitic compound IVM among 231 candidate small molecular compounds in the database of Drug Bank significantly reduced CTSB-induced NETs formation, with no effect on tumor cell viability. The prior investigation on repositioning drugs, such as mefloquine and albendazole have demonstrated to promote cancer treatment by targeting cell cycle arrest in melanoma cells (37, 38). IVM is widely used in both animals and human as an FDA-approved parasiticide (39). Except for its role as antiviral activity against several viruses such as coronaviruses, recent studies have revealed its role in various diseases, including sepsis, diabetes and human cancer disease (40–42). In tumor study, IVM has been identified to reverse the chemotherapeutic drugs resistance through EGFR pathway in colorectal and breast cancer (43). In addition, another study points to a repression of WNT-β-CATENIN/TCF transcriptional response by IVM and related macrocylic lactones in human colon cancer (42). So further studies are required for us to investigate the role of IVM on melanoma cells in the pathogenesis of tumor metastasis.

Our immufluorescent results showed IVM reshaped the tumor immune microenvironment with increased CD8^+^T cells and reduced MDSCs in lung of melanoma mouse model. The results suggest that IVM exhibit a strong potential for melanoma cancer to lung metastasis. Melanoma is recognized as one of the most immunogenic human cancer types that has a strong correlation between the infiltration of T cells in melanoma metastases (44, 45). In clinical phase III trial with 945 patients, the overall 5-year survival rate was 44% for anti-PD-1 (46). Therefore, a combination of anti-PD-1 antibody with IVM should be added to further explore their effect on melanoma cell metastasis. Besides the role of CD8^+^T cells in melanoma metastasis, MDSCs were also found to be enriched and activated in the melanoma microenvironment. The reduced frequencies and cell number of MDSCs abrogate immunosuppressive functions that delay the tumor progression and prolong the survival both in animal models and in cancer patients (47, 48). So further studies are required to investigate the direct effect on IVM on CD8^+^T cells and MDSCs in melanoma metastasis.

Our molecular mechanism study identified IVM directly interact with GSDMD and promotes GSDMD oligomerization in bone marrow neutrophils. GSDMD is a pore forming protein that acts as a downstream molecular of inflammasome and non-canonical inflammasome (caspase-11). In innate immune cells such as macrophage, GSDMD is activated by the cleavage of canonical (NLRP3, AIM2, and caspase-1) or noncanonical pathway. Once cleaved, GSDMD can translocate to the plasms membrane to form pores and induce a lytic proinflammatory of cell death (49, 50). However, unlike macrophages, which undergo pyroptosis, the activation of canonical inflammasome in neutrophils facilitate NETosis. Recently, two studies have identified that the activation of gasdermin D (GSDMD) is required for the generation of NETs (32, 33). Secondly, although many studies have been focused on the regulation pathway for GSDMD cleavage (such caspase-8, caspase-3). Little is known for the regulation role of GSDMD pore formation on the plasma membrane. Our results further confirm a role of IVM on GSDMD oligomerzization, which is essential for GSDMD pore formation (2, 51). However, our results showed no effect of GSDMD cleavage after IVM treatment. The upstream enzyme of GSDMD is caspase-1 and caspase-11, our results showed there is no comparable effect of caspase-11 after IVM treatment. Since caspase-1 was not required for GSDMD-dependent NETs (32). We did not detect the expression of caspase-1, especially in neutrophils, except for caspase-1 and caspase-11, the resident granzyme in neutrophil can also cleave GSDMD including elastase and cathepsin G (52, 53). So, our study should also investigate the effect of IVM on elastase and cathepsin G.

The results of immunofluorescence and scanning electron microscope showed the release of NETs were significantly increased after CTSB treatment. Indicating the role of CTSB on NETs formation. Except for the role of CTSB on NETs, it has been identified that cathepsin protease is essential for melanoma progression (54). Intracellular cathepsins cleave proteins while extracellular cathepsins degrade type I collagen and active pro-invasive proteases in the tumor microenvironment, which promotes tumor metastasis (55). CTSB cleaves in the non-helical telopeptide extensions of collagens (56). The secretion of CTSB is significantly increased in cancer cells and are active and stable in acidic environments and neutral acidity (57). A study showed that Abl/Arg promoted CTSB secretion through controlling the transcription factors with epithelial mesenchymal transition (EMT), invasion and therapeutic resistance in melanoma cells (58). Except for the role of CTSB on tumor cells, there are some reports that CTSB interacting with NLRP3 and to subsequent caspase-1 activation in macrophages (59). In our study, further investigations are thus needed to explore the role of CTSB on other cells.

It has been identified that inflammatory cytokines and growth factors play a crucial role in tumor growth and tumor microenvironment. The qPCR results showed that the level of proinflammatory cytokines including IL-1β, IL-6 and TNF-α were significantly reduced in lung of B16F10 mice after IVM treatment. Melanoma cells often express variable levels of IL-1β and IL-6, which plays an important role of cell proliferation and melanoma progression (60). Also, the level of TGF-β, VEGF and MMP9 were reduced after IVM treatment. TGF-β is a multifunctional cytokine belong to the transforming growth factor superfamily, the key function is to regulate the inflammatory process. In melanoma, TGF-β is considered a marker of metastatic spreading (61). In addition, recent studies suggested that TNF-α and metalloproteases were key players in melanoma cells aggressiveness (62, 63). So further studies are required to investigate the precise effect of IVM in these pro-inflammatory cytokines and growth factors as secreted from B16F10 melanoma cells.

Overall, the data from this study suggest the role of IVM in the formation of CTSB induced reticular formation when melanoma metastasizes to the lungs. We found that CTSB secreted by tumor significantly promoted the activation of neutral caspase-11/GSDMD cells IVM directed at SDMX has greatly inhibited SDMX oligarchy and the subsequent GSMX core network. As the clinical needs of patients with melanoma have not been met, it is necessary to further explore the feasibility of this drug as a treatment for melanoma metastasis.

## Date availability statement

The original contributions presented in the study are included in the article/supplementary material. Further inquiries can be directed to the corresponding authors.

## Ethics statement

The animal study was reviewed and approved by All animal experiments were approved by the Institutional Animal Care and Use Committee of Fudan University. The maximal tumor measurements/volumes are in accordance with the IACUC.

## Author contributions

HZ designed and performed experiments. XX helped with the experiments. RX and TY supervised the study and wrote the manuscript. All authors contributed to the article and approved the submitted version.

## Funding

This work was funded by The National Key Research and Development Program of China (Grant No. 2019YFF021650210).

## Conflict of interest

The authors declare that the research was conducted in the absence of any commercial or financial relationships that could be construed as a potential conflict of interest.

## Publisher’s note

All claims expressed in this article are solely those of the authors and do not necessarily represent those of their affiliated organizations, or those of the publisher, the editors and the reviewers. Any product that may be evaluated in this article, or claim that may be made by its manufacturer, is not guaranteed or endorsed by the publisher.
